# Gene expression profiling may improve diagnosis in patients with carcinoma of unknown primary

**DOI:** 10.1038/sj.bjc.6604315

**Published:** 2008-04-15

**Authors:** J Bridgewater, R van Laar, A Floore, L Van'T Veer

**Affiliations:** 1 University College London Cancer Institute, London WC1E 6DD, UK; 2 Agendia BV, Slotervaart Hospital 9D, Louwesweg 6, Amsterdam 1066 EC, The Netherlands

**Keywords:** carcinoma of unknown primary, CUP, diagnosis, tumour classification, gene expression profiling, microarray

## Abstract

Carcinomas of unknown primary (CUP) represent between 3 and 10% of malignancies. Treatment with nonspecific chemotherapy is commonly unhelpful and the median survival is between 3 and 6 months. Gene expression microarray (GEM) analysis has demonstrated that molecular signatures can aid in tumour classification and propose foster primaries. In this study, we demonstrate the clinical utility of a diagnostic gene expression profiling tool and discuss its potential implications for patient management strategies. Paraffin tumour samples from 21 cases of ‘true’ CUP patients in whom standard investigation had failed to determine a primary site of malignancy were investigated using diagnostic gene profiling. The results were reviewed in the context of histology and clinical history. Classification of tumour origin using the GEM method confirmed the clinicians' suspicion in 16 out of 21 cases. There was a clinical/GEM inconsistency in 4 out of 21 patients and a pathological/GEM inconsistency in 1 patient. The improved diagnoses by the GEM method would have influenced the management in 12 out of 21 cases. Genomic profiling and cancer classification tools represent a promising analytical approach to assist with the management of CUP patients. We propose that GEM diagnosis be considered when the primary clinical algorithm has failed to provide a diagnosis.

Patients with carcinoma of unknown primary (CUP) present with metastatic disease for which the primary site cannot be found despite standard investigation. The incidence of patients with CUP is estimated at 3–10% and despite recent improvements in diagnosis there remains a cohort of ‘true’ CUP patients. Most clinical algorithms rely on clinical investigation and immunohistochemistry (IHC) with a growing panel of antibodies of different tumour specificity (Hainsworth *et al*, 1991; Raber *et al*, 1992; Brown *et al*, 1997; DeYoung and Wick, 2000; Varadhachary *et al*, 2004). Persistent investigation outside of algorithms rarely leads to the detection of a primary site as well as being emotionally, physically and financially draining. Where algorithms fail, physicians should avoid the temptation to ‘guess’.

Patients with CUP constitute not only those elderly with poor performance status (PS) and multiple comorbidities but also those who respond to treatment, resulting in both improved quality of life and survival. Multiple empirical chemotherapy regimens have been used for CUP patients, but there are few randomised data to support a specific regimen (Briasoulis *et al*, 2005). The median survival in randomised studies is approximately 7 months (Abbruzzese *et al*, 1995), which is both poor and significantly less than the expected survival for patients with breast and bowel malignancy following standard therapy. These data suggest that little further benefit can be gained from the use of empirical regimen, perhaps because of the heterogeneity of tumour phenotype represented in the true CUP population. It follows that specific treatment programmes may be beneficial in specific patients if the diagnosis can be improved.

Recently, several independent studies have demonstrated proof of principle for the use of gene expression microarrays (GEMs) in identifying a foster primary site for CUP (Giordano *et al*, 2001; Ramaswamy *et al*, 2001; Su *et al*, 2001; Dennis *et al*, 2002; Buckhaults *et al*, 2003; Shedden *et al*, 2003; Bloom *et al*, 2004; Tothill *et al*, 2005) Data from GEM of known primary tumours have been examined ‘blindly’ and the correct primary site identified with up to 89% accuracy. The success of these studies demonstrates that patterns of gene expression remain consistent with tissue of origin, both in cell lines (Ross *et al*, 2000) and tumour samples (Khan *et al*, 2001). Tothill and co-workers have developed a data set using 229 cancers and used this to predict a foster primary in a potential CUP population. Predictions were made in 11 out of 13 cases analysed but these patients were in the early stages of the diagnostic process and the diagnosis may have been obtained without the help of molecular prediction. Although this represents a validate of the technique, these are not ‘true’ CUP patients.

The clinical application of using GEM analysis to direct patient management has not been demonstrated in prospective studies. A major hurdle has been the availability of fresh tumour tissue, as the preservation of diagnostic material is nearly always in formalin and paraffin. RNA isolation from these materials has been challenging, but significant improvements in RNA isolation protocols for archival formalin-fixed, paraffin-embedded (FFPE) tissues have been made in recent years. By exploiting these advancements in RNA isolation methods, Ma *et al* (2006) developed a microarray gene expression database of 466 frozen and 112 FFPE samples of both primary and metastatic tumours, representing 43 tumour types. Using this database, a *k*-nearest neighbour classifier was constructed, which was subsequently transformed into a high-throughput diagnostic CUP microarray test.

In this study, we describe the use of this diagnostic classifier to examine tumours of 21 CUP patients and compare the results with results from clinical and histological information. We describe the process, the clinical relevance of each result and the implications for patient management. We propose a strategy to advance the approach of tumour-specific management for patients with CUP.

## MATERIALS AND METHODS

### Study design

This was a retrospective analysis of 21 patients with CUP diagnosed between September 2000 and May 2006. Patients were diagnosed and treated at three centres in the North London Cancer Network and the laboratory analysis performed at a single centre (Agendia BV, Amsterdam, The Netherlands). Ethical approval was obtained for the study. As internal controls, three samples from one patient taken at different times and two samples from another patient taken from two different anatomical sites were tested.

### Patients

Eligible patients were those who had undergone standard investigation for CUP. Histologically or cytologically confirmed adenocarcinoma, poorly differentiated carcinoma and squamous cell carcinoma were permitted. There had been no primary site identified following complete history, physical examination, chemistry profile, computed tomography (CT) scan of the chest, abdomen, and pelvis, mammography in women, PSA in men and directed workup of any symptomatic areas. Patients in the following categories were excluded: women with adenocarcinoma involving only axillary lymph nodes or the peritoneal cavity, patients with squamous cell carcinoma involving only cervical lymph nodes or inguinal lymph nodes, patients with poorly differentiated carcinoma consistent with a germ cell tumour (isolated midline structures, multiple pulmonary nodules, or elevated levels of *β*-human chorionic gonadotropin or *α*-fetoprotein), men with prostate-specific antigen elevated in their plasma or stained in their tumour, patients with a single, small, potentially resectable tumour and patients with neuroendocrine carcinomas.

### Laboratory analysis

Three 4-μm paraffin sections per patient were submitted for RNA isolation and microarray analysis. One section from each tumour sample was stained with haematoxylin and eosin for histological evaluation, and RNA was isolated, amplified and labelled, as described previously (Ma *et al*, 2006). Purified amplified cRNA was then conjugated to Cy-mono NHS ester (GE Healthcare, Little Chalfont Bucks, UK). Cy5 is used for the experimental samples and Cy3 for the reference sample. The labelled cRNAs were subsequently hybridised to a customised eight-pack microarray (R van Laar, personal communication). After hybridisation, the slides were washed and subsequently scanned with a dual-laser scanner (Agilent Technologies, Mountain View, CA, USA).

### Data analysis

The diagnostic eight-pack array consists of eight mini arrays with 1900 features each, and these features comprise 255 positive controls, 100 negative controls and 1545 biological sequences used for data normalisation and sample classification. Selection and validation of biological sequences used for sample classification is described elsewhere (Ma *et al*, 2006). Fluorescence intensities, captured in the form of TIFF images, were quantified, corrected for nonspecific background hybridisation, and normalised using Agilent Feature Extraction software (Version 8.5.1; Agilent Technologies).

A five-nearest neighbour algorithm (Kuruvilla *et al*, 2002) is used to determine the five most molecularly similar tumours in the CupPrint database, which consists of a total of 643 samples representing 48 tumour types. The class labels (i.e., tumour type) of these ‘neighbouring’ samples are used to infer the tumour type of each test sample. In the case of multiple tumour types being present within the nearest five neighbours to a given test sample, the ranking and number of each class within these five positions are taken into consideration. The GEM result is associated with a prediction score. This value positively correlates with an increase in consistency of tumour types represented by the five-nearest neighbours of a given test sample. Information about patient gender and anatomical site of biopsy was provided. A prediction score was given that comprises weightings for the site of biopsy, gender and the five-nearest neighbour analysis.

### Clinical analysis

A case-by-case analysis of the patients was performed. Key outcomes were whether the GEM prediction was clinically feasible, agreed with clinical characteristics and histology results and whether the prediction would have changed management. A survival analysis was performed on the population and a prediction of survival based on median published survival time following tumour-specific treatment (Coleman *et al*, 2004).

## RESULTS

### Patient characteristics

Patient characteristics are given in Table 1. The median age of patients was 65 years, with 8 out of 21 patients below the age of 60 years. Generally, the clinical characteristics of the patients were typical for a CUP population. Out of 21 patients, 5 were of poor PS and unable to receive palliative treatment, but 11 out of 21 were of PS 0–1 and 9 out of 21 proceeded to palliative chemotherapy, the remainder being placed on surveillance. Out of 21 patients, 18 had a single site of metastasis. Although the sample may be considered unrepresentative because 18 patients had only one metastatic site, the median survival of this patient cohort was 4 months, consistent with a cohort of patients with a significant tumour load and a poor prognosis. The short median survival was influenced by five patients with poor PS not suitable for chemotherapy. The overall survival is consistent with data from randomised chemotherapy studies with median survival of 6–9 months (Pavlidis *et al*, 2003).

Patients with CUP in well-described subgroups (see eligibility) were excluded from our study, as they are often treated as for a probable primary site: a high neck node with squamous cell carcinoma histology is treated as a head and neck cancer despite a normal nasopharyngoscopy, and an axillary node with adenocarcinoma histology is often treated as breast cancer despite a normal breast. The survival of these subgroups is in most cases equivalent to that of the adopted primary cancers.

### Diagnosis and GEM results

Tumour origin predictions for samples in this study were obtained from paraffin-fixed tissue biopsies. Gene expression data of each sample were compared to the CupPrint database. Expression of 495 genes is quantified, and a prediction of tumour origin generated using a *k*-nearest neighbour algorithm is applied.

In Table 2, the GEM-predicted diagnosis, prediction score and five closest tumour types for each of the 21 patients of this study are listed. The predicted diagnosis is the most likely site of origin. The prediction score is an indication of the homogeneity in the top five closest tumour types, for example, samples with a high prediction score (>0.8) have at least four of the top five closest tumour types in common. The predicted site of origin is the tumour in the database to which the samples have the most similarity to.

For 14 out of 21 patients, the predicted diagnosis suggested a commonly occurring foster primary tumour (colon, breast and ovary). For 7 out of 21 patients, the predicted site of origin proposes a less common primary site. The historical median survival for those patients with common tumours with tumour-specific therapy would be expected to be greater than that documented for this population (Hurwitz *et al*, 2004; Robert *et al*, 2006).

In three patients, the clinical scenario was inconsistent with the GEM-predicted foster primary (Table 3). For case 16, the predicted site of origin was mesothelioma, which is an incorrect result, as adenocarcinoma had been identified histologically. For case 18, the predicted site of origin of endometrial carcinoma is unlikely, as the patient had a normal hysterectomy specimen the previous year. For case 19, the predicted site of origin of pancreatic adenocarcinoma is also unlikely, as the positron emission tomography (PET) and CT did not demonstrate a primary pancreatic site and the IHC result was not supportive of a pancreatic primary. In addition, the patient is alive and well 24 months after diagnosis, inconsistent with the median survival for patients with metastatic adenocarcinoma of the pancreas (6 months with chemotherapy; Burris *et al*, 1997).

### Reproducibility

There was a consistency between identical samples analysed independently. Three samples from case 3 submitted blind to the laboratory as three different patients resulted in identical reports. Analysis of two samples from different sites of tumour metastasis in the same patient resulted in the identification of the same origin as primary tumour, indicating accuracy and reproducibility of the test. This also means that the site of metastasis, in this case lymph node and skin, did not substantially affect the genomic profile of this sample.

### Management change

Potential changes in management are described. Twelve patients (57%) may have had a change in management if GEM prediction had been available at the time of diagnosis. For example, in patient 2, the clinical algorithm, including a negative mammogram and oestrogen receptor-negative tumour tissue, did not reveal the primary cancer (Table 3). However, if the diagnosis of breast cancer as primary cancer would have been known earlier, the patient could have been treated with breast cancer-specific therapy.

For nine patients, supportive care was the primary management, and improved diagnosis may not have led to management change in all of these patients. The diagnostic profiling provided a feasible clinical diagnosis yet a change of treatment would have been unlikely to change outcome in patients with poor PS, as patients with poor PS would be unlikely to receive and benefit from chemotherapy. A prospective study would more accurately identify the proportion of patients whose management would change following a GEM prediction.

## DISCUSSION

This study proposes the potential clinical use of an improved diagnosis for CUP patients. The primary findings are that a clinically feasible result was obtained in 18 out of 21 cases, that foster primaries were mostly (14 out of 21) common cancers and that the management would be influenced in 12 out of 21 cases.

Multiple chemotherapy empirical regimens have been used in CUP without clear advantage. The *a priori* explanation lies in the heterogeneity of tumours that may constitute a CUP patient population and lack of specificity of any empirical combination. Improved diagnosis and the use of specific regimen as for the common tumour types is clearly desirable.

The current standard of care employs IHC as an adjunct to conventional clinical and pathological investigations (Varadhachary *et al*, 2004). Despite a growing panel of antibodies, the success rate in identifying specific subgroups of CUP patients is only 20% (Pavlidis *et al*, 2003), although the use of a diagnostic algorithm is able to correctly identify the primary sites of known primaries in 88% (Dennis *et al*, 2005). Computed tomography scanning of abdomen and pelvis is very well documented and results in detection of a primary site in 30–35% of patients. With increasingly accurate diagnostic tools such as PET, endoscopic ultrasound and improved IHC, the likelihood of misdiagnosis is small. Nevertheless, there remains a core of patients for whom the primary is uncertain. Rades *et al* (2001) reported that PET allowed detection of the primary site in 43% (18 out of 42 patients) of the study population that included only localised CUP based on conventional staging procedures. Dissemination was detected by PET in 38%, and in 69%, the PET result influenced the selection of the definitive treatment. However, the false-positive rate of PET is approximately 20%, since this method is not specific for tumour tissue. Additionally, very small tumours cannot be seen with PET (for review, see Jerusalem *et al*, 2003).

Persistent investigation in search of the primary tumour is often time consuming and expensive yet futile. The total cost of all investigations for CUP patients was calculated to be between $4500 and 18 000 per patient (Schapira and Jarrett, 1995). It is possible that some of these costs could be reduced if molecular profiling would be used in the diagnostic process.

We propose that GEM diagnosis be considered when the primary clinical algorithm has failed to provide a diagnosis. The results from the genomic profiling should be weighed carefully with the clinical picture and discriminatory tools such as IHC.

We have presented the predicted primary sites for a cohort of patients with CUP using diagnostic profiling. The analysis suggests that the primary site can be predicted in the majority of patients, that this prediction is robust and that those cancers proposed are mostly common cancers. The hypothesis that therapy based on diagnostic GEM prediction provides a more targeted therapy with a consequent improvement in survival remains to be tested in future clinical studies.

## Figures and Tables

**Table 1:** Patient characteristics

*Age (years)*	
Median	65
Range	35–88
	
*Gender*	
Male/female	10/11
	
*Survival (months)*	
Median	4
Range	0.5 to 48+
	
*Stage*	
PS 0	4
PS 1	7
PS 2	5
PS 3–4	5
	
*Histology*	
Adenocarcinoma	6
Poorly differentiated adenocarcinoma	10
Undifferentiated carcinoma	2
Not available	3
	
*Lymph nodes (N)*	5
Mediastinal	1
Supraclavicular	1
Inguinal	3
	
*Viscera (N)*	15
Liver	5
Peritoneum	3
Pleura	2
Ascites	1
Testis	1
Skin	2
Ovary	1
	
*No. of metastatic sites*	
1	18
2	3
>2	0

PS=performance status.

**Table 2:** Results from diagnostic gene profiling: predicted site of origin, prediction score and five closest tumour types

**Patient**	**Predicted site of origin**	**Prediction score**	**Nearest neighbour 1**	**Nearest neighbour 2**	**Nearest neighbour 3**	**Nearest neighbour 4**	**Nearest neighbour 5**
1	Small-cell-lung cancer	1	Small-cell-lung cancer	Small-cell-lung cancer	Small-cell-lung cancer	Small-cell-lung cancer	Small-cell-lung cancer
2	Adenocarcinoma of the breast	0.40	Endometrial cancer	Adenocarcinoma of the breast	Adenocarcinoma of the ovary	Adenocarcinoma of the breast	Adenocarcinoma of the ovary
3	Adenocarcinoma of the breast	0.81	Adenocarcinoma of the breast	Adenocarcinoma of the breast	Adenocarcinoma of the breast	Adenocarcinoma of the breast	Adenocarcinoma of the prostate
4	Adenocarcinoma of the colon	0.80	Adenocarcinoma of the colon	Adenocarcinoma of the colon	Adenocarcinoma of the pancreas	Adenocarcinoma of the pancreas	Adenocarcinoma of the breast
5	Adenocarcinoma of the colon	1	Adenocarcinoma of the colon	Adenocarcinoma of the colon	Adenocarcinoma of the colon	Adenocarcinoma of the colon	Adenocarcinoma of the colon
6	Adenocarcinoma of the colon	1	Adenocarcinoma of the colon	Adenocarcinoma of the colon	Adenocarcinoma of the colon	Adenocarcinoma of the colon	Adenocarcinoma of the colon
7	Adenocarcinoma of the colon	0.59	Adenocarcinoma of the stomach	Adenocarcinoma of the breast	Adenocarcinoma of the colon	Adenocarcinoma of the colon	Adenocarcinoma of the colon
8	Adenocarcinoma of the small or large bowel	1	Adenocarcinoma of the colon	Adenocarcinoma of the colon	Adenocarcinoma of the colon	Adenocarcinoma of the colon	Adenocarcinoma of the colon
9	Adenocarcinoma of the small or large bowel	0.81	Adenocarcinoma of the small or large bowel	Adenocarcinoma of the small or large bowel	Liver cancer	Adenocarcinoma of the small or large bowel	Adenocarcinoma of the small or large bowel
10	Adenocarcinoma of the small or large bowel	1	Adenocarcinoma of the small or large bowel	Adenocarcinoma of the small or large bowel	Adenocarcinoma of the small or large bowel	Adenocarcinoma of the small or large bowel	Adenocarcinoma of the small or large bowel
11	Adenocarcinoma of the small or large bowel	1	Adenocarcinoma of the small or large bowel	Adenocarcinoma of the small or large bowel	Adenocarcinoma of the small or large bowel	Adenocarcinoma of the small or large bowel	Adenocarcinoma of the small or large bowel
12	Adenocarcinoma of the ovary	1	Adenocarcinoma of the ovary	Adenocarcinoma of the ovary	Adenocarcinoma of the ovary	Adenocarcinoma of the ovary	Adenocarcinoma of the ovary
13	Adenocarcinoma of the ovary	0.21	Adenocarcinoma of the ovary	Adenocarcinoma of the pancreas	Adenocarcinoma of the stomach	Adenocarcinoma of the small or large bowel	Bile duct and gall bladder cancer
14	Adenocarcinoma of the ovary	0.80	Adenocarcinoma of the ovary	Adenocarcinoma of the ovary	Adenocarcinoma of the ovary	Endometrial cancer	Adenocarcinoma of the ovary
15	Adenocarcinoma of the ovary	0.61	Adenocarcinoma of the ovary	Adenocarcinoma of the ovary	Adenocarcinoma of the ovary	Endometrial cancer	Adenocarcinoma of the ovary
16	Mesothelioma	0.39	Adenocarcinoma of the ovary	Adenocarcinoma of the pancreas	Mesothelioma	Mesothelioma	Adenocarcinoma of the stomach
17	Bladder cancer	1	Bladder cancer	Bladder cancer	Bladder cancer	Bladder cancer	Bladder cancer
18	Adenocarcinoma of the endometrium	0.60	Adenocarcinoma of the endometrium	Adenocarcinoma of the ovary	Adenocarcinoma endometrium	Adenocarcinoma endometrium	Adenocarcinoma of the ovary
19	Adenocarcinoma of the pancreas	0.82	Adenocarcinoma of the small or large bowel	Adenocarcinoma of the stomach	Adenocarcinoma of the pancreas	Adenocarcinoma of the pancreas	Adenocarcinoma of the stomach
20	Cholangiocarcinoma	0.80	Cholangiocarcinoma	Cholangiocarcinoma	Cholangiocarcinoma	Cholangiocarcinoma	Adenocarcinoma of the pancreas
21	Adenocarcinoma of the stomach	0.4	Adenocarcinoma of the stomach	Adenocarcinoma of the stomach	Bile duct and gall bladder cancer	Adenocarcinoma of the ovary	Cervical squamous cell carcinoma

**Table 3:** Suggested management change for patients after GEM analysis and likely change in outcome

**Patient**	**Working diagnosis**	**Actual treatment**	**GEM result**	**Change of treatment**	**Change to**	**Actual survival**	**Predicted survival**	**Likely change in outcome**
1	Sarcoma	ECF	Small-cell-lung cancer	Yes	Carboplatin Etoposide	8	9	No
2	Unknown	Supportive care	Adenocarcinoma of the breast	Yes	Doxorubicin Paclitaxel	7	24	Yes
3	Unknown	Surveillance	Adenocarcinoma of the breast	Yes	Surgery to breast	48+	24	Yes
4	Bowel	5FU	Adenocarcinoma of the small or large bowel	Yes	Oxaliplatin Fluoropyrimidine+	9	24	Yes
5	Unknown	Supportive care	Adenocarcinoma of the small or large bowel	No	No	2	24	No
6	Unknown	Supportive care	Adenocarcinoma of the small or large bowel	No	No	1	24	No
7	Unknown	ECF chemotherapy	Adenocarcinoma of the small or large bowel	Yes	Oxaliplatin Fluoropyrimidine+	7	24	Yes
8	Unknown	Supportive care	Adenocarcinoma of the small or large bowel	Yes	Oxaliplatin Fluoropyrimidine+	1	24	Yes
9	Unknown	Supportive care	Adenocarcinoma of the small or large bowel	No	No change	1	24	No
10	Unknown	Supportive care	Adenocarcinoma of the small or large bowel	No	No change	8	24	No
11	Unknown	Supportive care	Adenocarcinoma of the small or large bowel	Yes	Oxaliplatin Fluoropyrimidine+	7	24	Yes
12	Unknown	Supportive care	Epithelial ovarian cancer	Yes	Paclitaxel Carboplatin	33+	36	Yes
13	Ovarian cancer	CF chemotherapy	Epithelial ovarian cancer	Yes	Paclitaxel Carboplatin	0.5	36	Yes
14	Unknown	Cisplatin 5FU	Epithelial ovarian cancer	Yes	Paclitaxel Carboplatin	3	36	
15	Ovarian cancer	Carboplatin	Epithelial ovarian cancer	No	No change	33+	36	No
16	Bowel cancer	CF chemotherapy	Mesothelioma	No	NA	2	—	—
17	Unknown	Supportive care	Bladder cancer	Yes	Bladder-specific chemotherapy	0.5	14	Yes
18	Unknown	Supportive care	Endometrial cancer	No	NA	18+	12	—
19		Supportive care	Adenocarcinoma of the pancreas	No				
20	Lymphoma carcinoma	CHOP cisplatin	Cholangiocarcinoma	Yes	Cholangiocarcinoma-specific chemotherapy	24	12	Yes
21	Unknown	ECF	Adenocarcinoma of the stomach	Yes		3+	12	Yes

CF=cisplatin and fluorouracil; CHOP=cyclophosphamide, doxorubicin, vincristine and prednisolone; ECF=epirubicin, cisplatin and 5-fluorouracil; 5FU=5-fluorouracil; GEM=gene expression microarray; NA=not available.
